# A New Approach for Monitoring Ebolavirus in Wild Great Apes

**DOI:** 10.1371/journal.pntd.0003143

**Published:** 2014-09-18

**Authors:** Patricia E. Reed, Sabue Mulangu, Kenneth N. Cameron, Alain U. Ondzie, Damien Joly, Magdalena Bermejo, Pierre Rouquet, Giulia Fabozzi, Michael Bailey, Zhimin Shen, Brandon F. Keele, Beatrice Hahn, William B. Karesh, Nancy J. Sullivan

**Affiliations:** 1 Wildlife Conservation Society, Bronx, New York, New York, United States of America; 2 Vaccine Research Center, National Institute for Allergy and Infectious Disease, National Institutes of Health, Bethesda, Maryland, United States of America; 3 Departamento Biologia Animal (Vertebrados), Facultad de Biologia, Universidad de Barcelona, Barcelona, Spain; 4 ECOFAC, Libreville, Gabon; 5 AIDS and Cancer Virus Program, Frederick National Laboratory, Frederick, Maryland, United States of America; 6 University of Alabama at Birmingham, Birmingham, Alabama, United States of America; Centers for Disease Control and Prevention, United States of America

## Abstract

**Background:**

Central Africa is a “hotspot” for emerging infectious diseases (EIDs) of global and local importance, and a current outbreak of ebolavirus is affecting multiple countries simultaneously. Ebolavirus is suspected to have caused recent declines in resident great apes. While ebolavirus vaccines have been proposed as an intervention to protect apes, their effectiveness would be improved if we could diagnostically confirm Ebola virus disease (EVD) as the cause of die-offs, establish ebolavirus geographical distribution, identify immunologically naïve populations, and determine whether apes survive virus exposure.

**Methodology/Principal findings:**

Here we report the first successful noninvasive detection of antibodies against Ebola virus (EBOV) from wild ape feces. Using this method, we have been able to identify gorillas with antibodies to EBOV with an overall prevalence rate reaching 10% on average, demonstrating that EBOV exposure or infection is not uniformly lethal in this species. Furthermore, evidence of antibodies was identified in gorillas thought previously to be unexposed to EBOV (protected from exposure by rivers as topological barriers of transmission).

**Conclusions/Significance:**

Our new approach will contribute to a strategy to protect apes from future EBOV infections by early detection of increased incidence of exposure, by identifying immunologically naïve at-risk populations as potential targets for vaccination, and by providing a means to track vaccine efficacy if such intervention is deemed appropriate. Finally, since human EVD is linked to contact with infected wildlife carcasses, efforts aimed at identifying great ape outbreaks could have a profound impact on public health in local communities, where EBOV causes case-fatality rates of up to 88%.

## Author Summary

Ebolavirus causes deadly outbreaks in wild great apes, and has been reported as a significant threat to the survival of wild lowland gorillas in Central Africa. Improved knowledge of basic information regarding geographic distribution of ebolavirus in great ape populations, including the identification of immunologically naïve populations and the determination of whether apes survive virus exposure, will be needed in order for protective interventions such as immunization to be effective. However, monitoring ebolavirus infection in wild gorillas by current methods is challenging because of the difficulty in obtaining diagnostic samples from these elusive primates. Additionally, there are limitations associated with the available laboratory assays used to document ebolavirus infection. Here we report the first successful noninvasive detection of EBOV immunity in wild great apes, demonstrating survival in this species. This tool will be useful in a comprehensive strategy aimed at the protection of this endangered species and improved prevention of EVD outbreaks in human populations.

## Introduction

Emerging infectious disease (EID) epidemics and pandemics arise without warning, even with global efforts aimed at tracking pathogens early and at the source, a fact most recently evidenced by the swift global spread of influenza H1N1 [1], [2] and a current outbreak of ebolavirus affecting multiple West African countries simultaneously [3]. Most major human EIDs are of zoonotic origin and include viral infections of both global (HIV-1, HIV-2, H1N1) and localized significance (ebolavirus, monkeypox, Marburgvirus, Nipah virus, severe acute respiratory syndrome [SARS]-associated coronavirus) [2]. Systematic monitoring of people and wildlife at hotspots of EID is one strategy for preventing human pathogens of animal origin from reaching a pandemic state [4]. By detecting animal pathogens before or just as they emerge in humans, it may be possible to mitigate against their worldwide spread [2]. Furthermore, in the case of some diseases such as Ebola virus disease (EVD), the monitoring of wildlife disease serves as a critical component of early warning systems aimed at preventing the transmission of zoonotic diseases to humans [5], [6]. EVD has repeatedly passed from infected apes to hunters, leading to multiple epidemics and 360 human deaths (463 cases) in Gabon and the Republic of Congo (RoC) alone since 1994 [5], [7]–[9]. More significantly, human epidemics are often preceded by observed animal outbreaks, underlining the human health implications of surveillance and control of epizootics [5], [6].

The International Union for Conservation of Nature (IUCN) currently lists the western lowland gorilla (*G. gorilla gorilla*) as critically endangered and cites infectious disease as one of the top two threats to this species [10]. Ebolavirus is lethal in humans and nonhuman primates and has been described as a significant threat to the survival of western lowland gorillas and chimpanzees (*Pan troglodytes*) in Central Africa [7], [10], [11]. Data from ecological surveys in Central African ape habitats illustrate declines in ape signs (nests, feces, prints) temporally and spatially linked with confirmed human EVD outbreaks [12]–[14]. Mathematical modeling suggests that, between 1983 and 2000, gorilla numbers in Gabon dropped by more than 56%, and it is hypothesized that infectious pathogens, including ebolavirus and *Bacillus anthracis*, may contribute to gorilla mortality in Africa [10], [12], [15].

Despite the significance to both human and wildlife health, direct evidence of great ape exposure to ebolavirus or other pathogens (either by pathogen or immune response detection) is scant, complicating our ability to monitor epizootics. Therefore, to fill this gap, there is a need for prospective epidemiologic studies combining ecological data with laboratory screening. Most currently available data regarding primate pathology and immune response comes from experimentally infected laboratory macaques [16], [17].

In direct response to the challenges associated with collecting blood or tissue from wildlife, non-invasively collected biological samples such as feces have been used for wildlife disease screening [18], [19]. Primate feces have been screened for the presence of viral nucleic acids due to shedding of simian immunodeficiency virus (SIV), circoviruses, enteroviruses and hepatitis viruses [20]–[23]. For SIV, feces have also shown the presence of virus-specific antibodies [23]. We developed a non-invasive immunological assay to detect ebolavirus antibodies in great ape feces, allowing us more insight into wild ape ebolavirus infections and their surveillance, and leading the way to identifying the best approaches for their protection. In addition, this new assay may prove valuable in the development and employment of prospective epidemiological ebolavirus studies in wild great ape populations.

## Materials and Methods

### Site selection

Eighty gorilla fecal samples were collected in 5 different habitats in the RoC. In zone A, 20 gorilla fecal samples were opportunistically collected while following habituated gorillas roughly 2 and 3 years after ebolavirus infection was confirmed in ape carcasses at that site using a combination of RT-PCR, immunohistochemistry and antigen capture [5], [9].

In June 2007, 15 samples were collected in zone B1 during a reconnaissance walk survey (recces) composed of eight ∼10 km linear recces radiating every 30° from a central point with terminal ends of every other pair connected by 8 km recces. This zone is southwest of the Mambili River in the southeastern-most area of Odzala-Kokoua National Park (OKNP), and samples were collected two years after two gorilla carcasses found in this area tested positive for EBOV using RT-PCR and antigen capture assays [9], [24]. For these surveys, two teams operated simultaneously, each averaging 5.6 km per day over 5 days, and following pre-determined global positioning system (GPS) points. A continuous GPS track log was maintained and uploaded to a Garmin 12XL GPS (www.garmin.com) with a position recorded every 1 km.

Three missions occurred in zone B2. The first occurred from 30th August to 8th September 2005 when a 45 km closed loop survey was conducted on the northeast side of the Mambili River. This search was for evidence that would indicate that the above-described May 2005 epizootic southwest of the Mambili River had also affected wildlife on the opposite side of the waterway. Ten gorilla fecal samples were collected and a continuous GPS track log was maintained and uploaded to a Garmin 12 XL unit, with points taken every 5 km. Also, in 2005, a large-scale ecological and large mammal survey was conducted throughout OKNP under the auspices of the Wildlife Conservation Society and the *Projet Espèces Phares* of the European Union [25]. From September 5^th^ to 11^th^, 2005, five gorilla fecal samples were collected during these missions by means of reconnaissance walk surveys and of a systematic unbiased line transect design aimed to estimate animal abundance derived from the density of animal sign, multipliers decay rate and production, and the area of the survey zone; both designed with and analyzed by the Distance software program [26]–[28]. Lastly, in June 2007, the original 45 km loop described above was repeated during which 5 gorilla fecal samples were collected.

In November–December 2007 (Zone D) and March–April 2008 (Zone C), reconnaissance walk surveys, similar to the approach applied in zone B1, were conducted in great ape habitats that, by the end of the study period, had no reported disease outbreaks. The purpose of these missions was to estimate ape abundance by recording all ape nests. GPS points were taken every 5 km and 25 samples were collected.

### Sample collection and preservation

Sample collectors wore disposable latex gloves and surgical masks while collecting feces. Approximately 20 g of fresh feces was placed in 20 ml of RNAlater (Qiagen GmbH, Hilden, Germany) in a 50 ml plastic screw-top vial (Corning Incorporated, Corning, New York, USA), sealed with Parafilm (Pechiney, Menasha, WI, USA), and placed in zip-closure plastic bags and stored at ambient temperature (∼28°C or 82°F). Samples collected in zone B1 were placed in liquid nitrogen vapor in a dry shipper (Arctic Express Dual 10, Thermolyne) at the end of each day and maintained in this state until arrival at the analyzing laboratory. Feces were determined to be that of gorillas when recovered under one of the following conditions: post-observation collection (after seeing gorillas) or post-audition collection (after hearing gorillas), in association with gorilla nests or in association with gorilla trails [29], [30]. Genotype studies have demonstrated that feces collected using these methods are accurately classified as gorilla feces 98% of the time [30]. In addition, the presence of long tri-lobed sections, ample fiber, and abundant green leafy material further classified these samples as gorilla dung [31], [32]. Only feces estimated to be less than 24 hours old using published criteria [32] were collected.

### NP antigen preparation

The plasmid encoding EBOV NP is a p1012 derivative [16]. To purify the recombinant viral protein, plasmid p1012NP was tagged at the C-terminus by site-directed mutagenesis with the QuickChange XL Site-directed Mutagenesis kit (Stratagene, La Jolla, CA, USA). p1012NP was provided with the hexa-histidine tag. The tagged plasmids were transfected into human embryonic kidney cells (FreeStyle 293-F Cells, Catalog No. R790-07) obtained from Invitrogen (Carlsbad, CA) and grown in a shaking flask at 37°C under 8% CO_2_ with FreeStyle 293 Expression Medium (Invitrogen). The EBOV His-tagged NP was purified by nickel-affinity gel, Ni Sepharose 6 Fast Flow (GE Healthcare, Piscataway, NJ), and eluted with 400 mM imidazol. The concentration of purified NP protein was measured with Quick Start Bradford Protein Assay reagent (Biorad, Hercules, CA, USA) and used in the western blot assay.

### Western blot assays

To screen nonhuman primate fecal samples for ebolavirus antibodies, we adapted an existing enhanced chemiluminescent western blot assay [23]. Feces were vigorously mixed in RNAlater (Ambion Life Technologies, Grand Island, NY, USA), 1.5 ml of the mixture diluted in 7.5 ml of PBS-Tween-20 (0.05%), heated at 60°C for 60 minutes, centrifuged at 3500×g for 20 minutes and dialyzed in PBS 1X with stir bar at 4°C for 18 to 24 hours to resuspend fecal immunoglobulins that normally precipitate in RNAlater. Purified or cell lysate NP protein was denatured in Sample Reducing Agent (Invitrogen NuPAGE), heated at 70°C for 10 minutes, separated by 4–12% gradient sodium dodecyl sulfate polyacrylamide gel electrophoresis (SDS-PAGE) (0.25 µg per well) (Invitrogen NP0321, Carlsbad, California, USA), and followed by transfer to nitrocellulose membrane (Invitrogen LC2001, Carlsbad, California, USA) which was blocked with 5% nonfat milk in PBS-Tween (0.3%) and bovine albumin (2.4%). Membranes were then cut into strips and incubated overnight in fecal extract on a rocking plate. Specific NP-bound antibody was detected with goat-anti-human IgG peroxidase conjugate and the blot was visualized using an enhanced chemiluminescence detection system. The films were exposed to the immunoblot strips then scanned using an Epson Perfection 4870 Photo scanner. To define a cut-off of positivity we used the Image J program (ImageJ, NIH, Bethesda, Maryland, USA) that allowed us to subtract the background in each strip and to compute the integrated density of the band that is the sum of the values of the pixels in the selection in the blot. Specimens which showed no visible specific band in the blots were scored as negative whereas those which showed specific band (reactivity with a protein of approximate molecular mass of 115 kDa corresponding to EBOV nucleoprotein NP) were regarded as positive if their integrated density was in excess of the mean of integrated density plus 3 standard deviations of the negative blots. Blots with a weak specific visible band and an integrated density below this cutoff were classified as uncertain. A subset of samples collected in 2005 (the year of the last EVD outbreak in the region) was also screened for the presence of filovirus RNA using a nested RT-PCR. Fifty nanograms of total RNA isolated from RNAlater preserved fecal samples were extracted using the RNAqueous 4PCR kit (Ambion Life Technologies, Grand Island, NY, USA) and used in a one-step RT-PCR, followed by a nested PCR step. We used degenerate primer pairs in order to amplify a 245 bp fragment of the L polymerase gene from any Filovirus. The one step RT-PCR primers are: 5′- ATMGRAAYTTTTCYTTYTCATT-3′ and 5′RYTATAAWARTCACTRACATGCAT-3′; the nested PCR primers are 5′-TTYCCWAGYAAYATGATGGT-3′ and 5′- GGDATTRDRWARTGCATCCA-3′.

To assess the quality of the total RNA from the fecal sample, we amplified a housekeeping gene for each sample, the ß-glucuronidase gene (GUS, Accession number AF084552) using a nested PCR assay. The GUS primers used for the one step RT-PCR were 5′-GCTTACCACCCAGTTTGAG-3′ and 5′-TGGGGATACCTGGTTTCATTG-3′, whereas the nested primers were 5′-TCAGAGCGAGTATGGAGC-3′ and 5′-GCACTTTTTGGTTGTCTC-3′. We generated a 253 bp fragment. Positive and negative controls were included to ensure that cDNA product could be amplified and that no contamination from cDNA or previous PCR products occurred.

### Statistical analysis

We compared antibody prevalence between sampling locations using a log-likelihood ratio test (G-Test) [33]. A 95% confidence interval (CI) was constructed for the prevalence.

## Results

### Detection of ebolavirus antibodies in fecal samples

In order to examine ebolavirus exposure in wild great apes we sought to develop a strategy of detection in samples collected by non-invasive methods that would be sensitive and specific enough to detect multiple ebolavirus species with minimal false positive results. It has been shown previously that an enhanced chemiluminescent western immunoblot assay is able to successfully detect specific antibodies in RNAlater-preserved feces from simian immunodeficiency virus-infected chimpanzees (SIVcpz) [23]. The sensitivity and the specificity of SIVcpz antibody detection in fecal samples were estimated to be 92% and 100%, respectively. Viral SIVcpz nucleic acid could be amplified in an immunoblot-positive fecal sample, confirming SIVcpz infection [23]. Furthermore, a similar approach was used to diagnose simian foamy virus infection in wild chimpanzees (SFVcpz). The sensitivities of SFVcpz antibody and viral nucleic acid detection in fecal samples from captive chimpanzees were 73% and 75% respectively, and assay specificities were 100% [34]. These studies show the potential of assessing RNAlater-preserved fecal samples to document wild apes' exposure to viruses.

Given the success of this approach, we developed a fecal western blot assay to detect ebolavirus antibodies. We chose purified EBOV NP as the antigen for antibody detection since it is one of the most abundant structural proteins produced during infection and a major target of the host immune response. This is supported by previous studies showing that humans who have survived natural EBOV infection developed strong antibody responses mostly against NP [35]–[37]. In addition, the NP sequence is well conserved among ebolavirus species (Figure S1), making it useful for detection of antibodies against multiple ebolavirus species [38], [39] and potentially increasing the breadth of this detection method.

We first assessed the ability to detect NP antibodies in RNAlater-preserved fecal samples from captive cynomolgus macaques. Fecal specimens were experimentally spiked with different dilutions of positive serum containing polyclonal immunoglobulin from a monkey that was vaccinated with a genetic vaccine encoding NP [16]. Serum from this vaccinated monkey displayed antibody reactivity with NP by both ELISA and western blot analysis (not shown).

**Figure 1:**
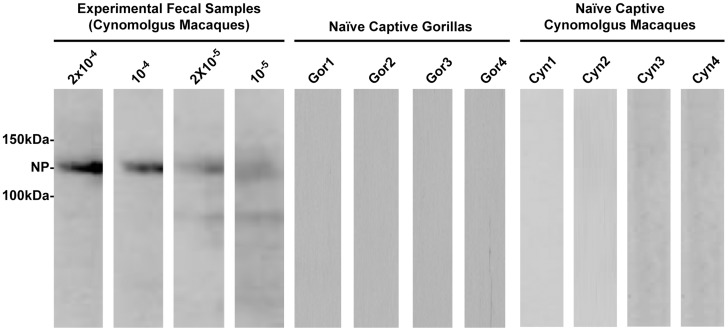
Western blot detection of ebolavirus antibodies in fecal samples. The experimental limit detection of the assay was determined by probing immunoblots with fecal extracts from experimental positive fecal samples. Western blot of immobilized NP strips showing dilutions of cynomolgus macaque fecal extracts spiked with polyclonal serum containing NP-specific antibodies (left four lanes). Representative blots of negative fecal extract controls from naïve captive gorillas (Gor1-Gor4) and uninfected naïve captive cynomolgus macaques (Cyn1-Cyn4) were included to assess specificity. The approximate molecular weight of NP is indicated.

Extracts from these positive serum-spiked feces were then used to incubate immunoblot strips containing immobilized NP. Anti-NP antibodies were detected by enhanced chemiluminescent western blot immunoassay in fecal samples at seropositive nonhuman primate (NHP) plasma dilutions of up to 10^5^-fold (Figure 1), indicating a high sensitivity of the assay for fecal antibody detection. A similar level of sensitivity was observed for detection of anti-SIV and anti-HIV antibodies by western immunoblots using plasma samples from SIVsm-infected NHP diluted up to 10^−4^ and plasma samples from HIV-1 infected individual diluted up to 10^−6^ [40]. In contrast, fecal extracts from captive and uninfected nonhuman primates (cynomolgus macaque and western lowland gorilla species) treated in the same way showed no reactivity in the NP immunoblot, demonstrating low background for the assay and lack of cross-reactivity with serum antibodies directed against irrelevant proteins. These results demonstrated that NP antibodies present in primate fecal samples can be extracted and detected by immunoblotting.

### Survey of wild apes

To evaluate whether wild apes show evidence of previous ebolavirus exposure, we screened 80 fecal samples from gorillas living in the RoC for ebolavirus antibodies. Fecal samples were opportunistically collected from great ape habitats using one of two survey methodologies. The first method employed a systematic unbiased line transect design aimed to estimate animal abundance or the density or size of wildlife [26]–[28]. The second consisted of reconnaissance walks to provide a general overview of large animal distributions and investigate animal trails where animal dung is likely to be encountered [41].

**Figure 2:**
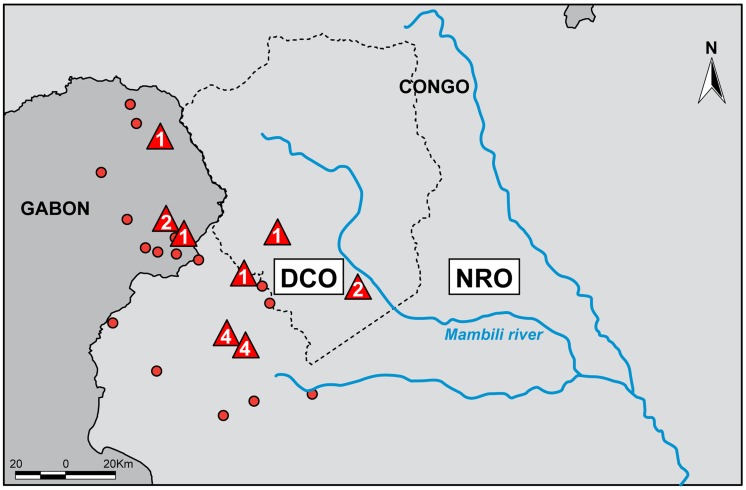
Confirmed human and ape Ebola virus (EBOV) infection in relation to sampling zones. Numbers in red triangles correspond to the number of great ape carcasses previously reported to be positive for EBOV infection by more than one diagnostic test, which includes antigen detection, DNA amplification or immunohistochemical staining. Red circles represent villages with recorded human EBOV outbreaks. Blue lines are rivers, and the limits of the Odzala-Kokoua National Park are shown by a dashed gray line. DCO: diagnostically confirmed outbreak region (south and west of the Mambili River). NRO: no reported outbreak region (north and east of Mambili River).

Fecal samples were collected from two regions within or adjacent to OKNP in western RoC near the border with Gabon (Figure 2). The first is an EVD diagnostically confirmed outbreak (DCO) region where human cases were laboratory confirmed between 2001 and 2005 [13], and ape carcasses collected between 2002 and 2005 tested positive for EBOV [5], [9], [24]. The presence of long-term and functioning wildlife disease surveillance programs and gorilla habituation and research studies in the RoC allowed for immediate access to the DCO region during and after EVD epidemics which facilitated the collection of 35 samples from gorillas with a high likelihood of previous exposure to EBOV, and samples were collected at this site within 25–43 months of confirmed great ape EVD cases being found. The second region is an area with no reported outbreaks at that time (NRO). Here, there were no reported human cases, observable signs of epidemics, EBOV-positive animal samples, or significant losses in ape numbers despite repeated visits up until the end of this study in April 2008. Routine and systematic reconnaissance missions for ecological surveillance activities were used for the collection of 45 samples in the NRO region (Table 1).

**Table 1:** Prevalence of ebolavirus antibodies in gorilla fecal samples by sampling zone in the Republic of Congo (2005–2008).

					Zone		Region	
Region	Zone	Mission	Date collected	# Samples tested	# Positive/total tested	% Positive (95% Cl)	# Positive/total tested	% Positive (95% Cl)
DCO	A	Following ape groups	Aug–Sept 2005	20	2/20	10 (0–26.3)	5/35	14.2 (8.0–28.6)
	B1*	Survey 2 years post epizootic	June 2007	15	3/15	20 (0–47.9)		
NRO	B2	Closed loop survey	Aug–Sept 2005	10	3/20	15 (0–34.3)	3/45	6.6 (0–15.8)
	B2†	Large scale mammal survey	Sept 2005	5				
	B2	Revisit closed loop survey	June 2007	5				
	C	Ape survey 1	March 2008	11	0/15	0 (0–15.5)		
	C	Ape survey 2	April 2008	4				
	D	Ape survey 3	Nov–Dec 2007	10	0/10	0 (0–21.7)		
						**Total**	8/80	10 (3.1–17.7)

Numbers in parentheses are 95% Adjusted Wald confidence intervals.

DCO = Diagnostically confirmed outbreak.

NRO = No reported outbreak.

*Two uncertain western blot samples were counted as negative.

† One uncertain western blot sample was counted as negative.

### Identification of ebolavirus antibodies in wild great apes

**Figure 3:**
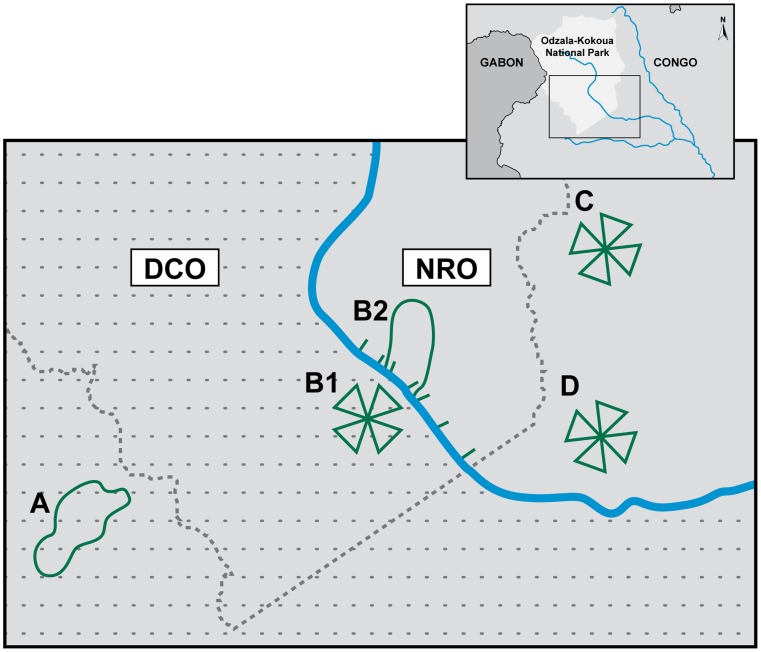
Sampling survey zones. Upper right panel: the region in Africa from which samples were collected. Large panel: enlargement showing collection zones; samples were collected within or adjacent to the Odzala-Kokoua National Park in western RoC near the border with Gabon and the large panel indicates the details of the fecal sample collection zones: A and B2 are fecal sampling zones using closed loop survey or line transects (green line) and B1, C and D are sampling zones by reconnaissance walk survey (triangle shapes) as described in Materials and Methods. The blue line is the Mambili River. The diagnostically confirmed outbreak region (DCO) south and west of the river is in gray with dash lines and the no reported outbreak (NRO) region north and east of the river is in gray without dash line. The border of the Odzala-Kokoua National Park is identified by a dashed gray line.

**Figure 4:**
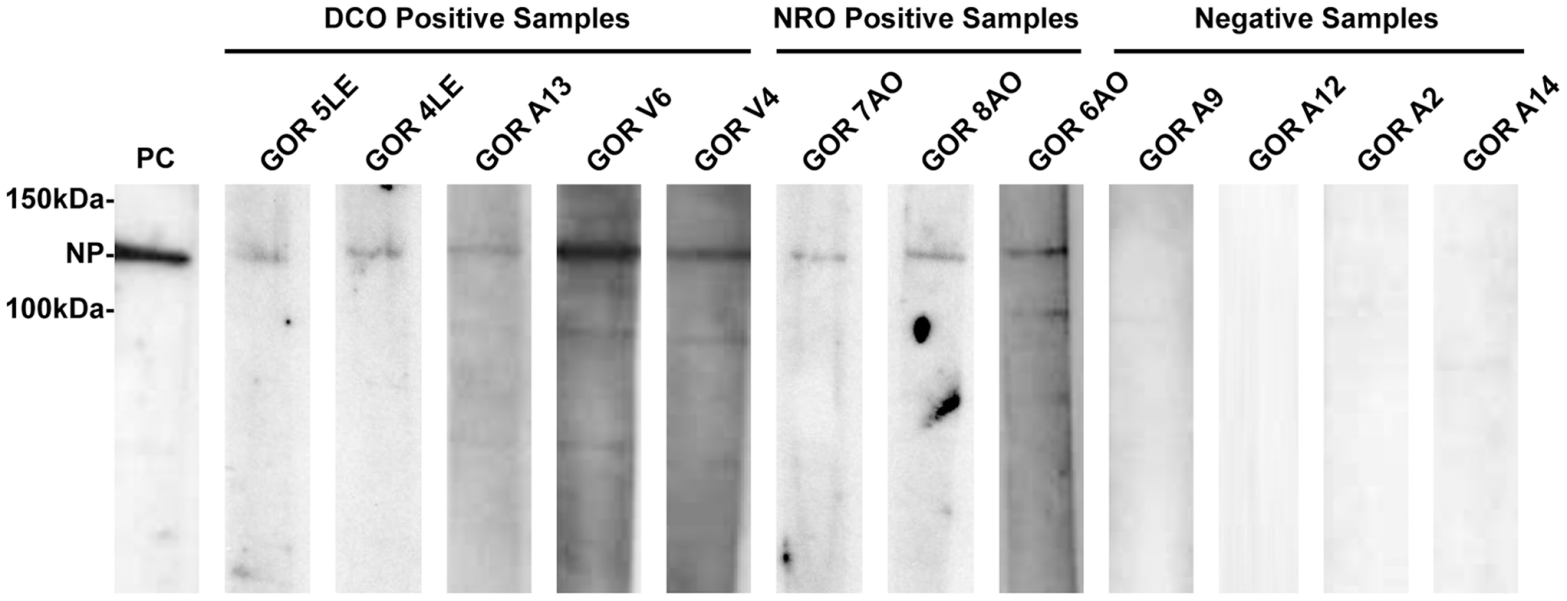
Detection of ebolavirus NP antibodies in gorilla fecal samples. Fecal samples from wild gorillas were tested by enhanced chemiluminescent western blot using strips containing immobilized Ebola virus NP. Positive samples are grouped according to the two collection regions, diagnostically confirmed outbreak (DCO) and no reported outbreak (NRO). Strips from representative negative samples are shown. The approximate molecular weight of NP is indicated. An experimental cynomolgus macaque fecal sample spiked with NP-positive serum was used as a positive control (PC).

All human EVD outbreaks that had previously occurred in our sampling zone study are thought to be the result of handling infected wild animal carcasses, including gorillas [13]. Samples from carcasses were used to document EBOV outbreaks in gorillas by RT-PCR, antigen detection ELISA and immunohistochemistry [5]. To explore whether ebolavirus antibodies could be detected in fecal samples obtained from wild apes we focused initially on the DCO region (zones A and B1; Figure 3) in order to maximize the likelihood of obtaining fecal samples from apes that had been exposed to EBOV. Among 35 fecal samples collected from the DCO region, 5 tested positive for NP antibodies by immunoblot (Figure 4, Table 1). Two EBOV antibody positive fecal samples out of 20 (10%, CI: 0–26.3%) came from zone A where, in late 2002 and early 2003, EBOV was laboratory confirmed in great ape carcasses at the Lossi Sanctuary [5], [24] (Figure 2). Of 15 samples collected in zone B1 in 2007, two samples were uncertain (defined in Methods) and three were positive for NP antibodies (23.3%, CI: 0–47.9%). Samples were collected from zone B1 during a mission two years after ebolavirus was detected in ape carcasses at the site [5], [24] (Figure 2).

### Great ape seropositivity in outbreak-free zones

We also tested ape fecal samples obtained from the outbreak-free (NRO) region to explore whether NP antibody detection can be used as a potential surveillance tool. The NRO region contains zones B2, C and D. Zone B2 is adjacent to B1, yet separated from it by the relatively large Mambili River. In the fall of 2005, fifteen ape fecal samples were collected in zone B2 to determine whether a May 2005 epizootic had also affected wildlife on the opposite side of the waterway. Two years later, in June 2007, the original 45 km closed loop track was repeated to explore any temporal changes in ape density or NP seropositivity. Three positive fecal samples out of twenty (15%, CI: 0–30.6%) were found in zone B2 (Table 1) and one sample was uncertain. Twenty-five fecal samples were collected in zone C (March and April 2008) and zone D (November and December 2007). The zone C mission followed the discovery of one chimpanzee carcass that later tested negative for EBOV (E. Leroy, personal communication, April 30, 2008). No antibodies were found in the fecal samples from zones C and D, where no outbreaks had been reported.

Altogether, eighty fecal samples from wild great apes were analyzed by Western blot and eight (10%) were found to be NP antibody positive (Table 1). Three samples (one from zone B2 and 2 from zone B1) had blots with a weak specific visible band and an integrated density below the cutoff, and were thus classified as uncertain (not shown). The remaining 69 fecal samples showed no detectable NP-specific antibodies and were classified as antibody negative. Roughly half of the samples were collected in the DCO region and 14.2% of these samples were found to be antibody positive, whereas a smaller proportion (6.6%) of samples collected in the NRO region were positive. The difference between the NRO and DCO regions is not statistically significant (log likelihood ratio statistic (G) = 1.3925, X-squared df = 1, p-value = 0.238) (Table 1), but overall the data show that anti-NP antibodies are present in fecal samples from wild ape populations even in areas with no prior reports of human or wild great ape outbreaks. These data demonstrate that the screening of wild gorilla feces by western blot for the purpose of monitoring ebolavirus exposure was successful in detecting NP antibodies.

## Discussion

This study represents the first time that ebolavirus antibodies have been detected in wild great ape fecal samples, and carries important implications for the future management and survival of these primates. This is especially relevant because intervention strategies to protect apes against future EVD infections are being actively explored, including vaccination since ebolavirus vaccines have been shown to protect laboratory monkeys from disease [42], [43].

There have been no studies or observations involving great apes that have described immune response, clinical signs, precise mortality rates or whether survivorship provides long-term immunity, and little is known regarding the overall ebolavirus serological status of apes in Central Africa. To date, serum samples from gorillas (n = 30) and chimpanzees (n = 256) in Central Africa have been screened for antibodies against EBOV [7]. Most of these animals were sampled while living in captive settings (pets, rescue centers, primate centers); four subjects were free-ranging and sampled directly from the wild in OKNP but were seronegative. Obtaining samples from free-ranging wildlife is needed to improve our understanding of infectious agents circulating in the environment.

All other health data related to ebolavirus from free-ranging apes comes from necropsies performed during wildlife die-offs and, in those cases, the vast majority of samples collected are too degraded to have diagnostic value [5]. As expected, there is also nothing known regarding potential co-infections involved in great ape EVD which may modify the host immune response, alter pathogenesis, increase mortality or influence the effectiveness of any future prophylactic plans, such as the administration of a vaccine once available. This is due to the difficulty in acquiring diagnostic samples from wild populations. Capture and subsequent blood collection for serological screening is costly, time consuming, and carries some risk to the animals while providing information on only a few individuals. In fact, despite the disappearance of a staggering number of great apes in Gabon and the RoC and 6 years of sustained and active surveillance in these countries during the course of this study, only 37 carcasses were recovered, with confirmed EBOV infection in 16 of those individuals [9]–[11],[24]. Moreover, finding animal carcasses in vast tracts of rain forest is difficult; it requires intensive searching and often results in the acquisition of highly degraded samples, which are not suitable for detection of viral antigens or nucleic acid and are more prone to negative results [5].

This newly developed approach for non-invasive sampling of great apes has allowed the successful detection of anti-EBOV antibodies in fecal samples, yielding a seroprevalence rate of 10% in gorillas. Since genetic identification of individual fecal samples was not performed, we cannot rule out the possibility of resampling, so the prevalence rate is an upper limit for this data set. However, recent genetic analysis of gorilla and chimpanzee samples collected during iron-cross recces (the type of surveillance executed in sites B1, C, and D) from 2006–2010 suggest a low resampling rate. Of 162 samples, three were identified with genetic identity the same as three previously sampled individuals, yielding a 2% resampling rate for sites in which the same site was revisited with the shortest interval of seven-months apart; the resampling rate in the currently study could be higher because two sites were sampled one-month apart and two locations, A and B2, were not iron-cross recces (personal communication, K.J. Lee)

In addition to estimating ebolavirus exposure in NHP, this technique of screening feces by western blot is in fact a multi-purpose tool. It provides the potential to employ serial fecal collections to detect a temporal change in incidence exposure in a given zone. For instance, we saw a trend toward a decrease in ebolavirus fecal antibodies in zone B1/B2 from 20% in 2005 to 12% in 2007, which can be tested in the future using formal prospective studies. Fecal antibody screening can also be used before and after vaccination to demonstrate vaccine-induced immune responses developed in great ape populations, noting that antibody levels in vaccinated non-human primates are an immune correlate of protection [42]. Finally, this approach will facilitate the identification of immunologically naïve populations for large-scale vaccination trials, thereby improving cost-effectiveness by identifying communities that could benefit the most from vaccination efforts. Along these lines, it provides us with the first real possibility to investigate patterns of EVD emergence in wild apes independent of animal mortality and the role natural barriers, such as rivers, may have in mitigating its spread. This ability to map exposure patterns across Central Africa may also provide insight into how this virus spreads within and between ape populations, a question that has generated two disparate theories: multiple virus introductions and a single spreading outbreak [10], [11], [24], [44].

Key to pandemic prevention is disease surveillance at the human/wildlife interface, especially considering the fact that the majority of emerging infectious diseases events (over 60%) are of animal origin and that those caused by wildlife pathogens are increasing [6], [45]. The strategy described herein will be valuable in providing zoonotic information of public health concern from regions where resources are poor and help counter the emergence of diseases which have potential to become the next pandemic. Monitoring diseases in animals using methods such as those we describe here allows for the identification and surveillance of many pathogens, including those with potential to adapt and spread in humans, like HIV and plasmodium parasites [23], [46], [47]. These findings also illustrate the role *in situ* conservation organizations can play in disease surveillance programs.

Adapting these tools for use in other wildlife species may provide information regarding the transmission of ebolavirus and other emerging infectious diseases to human populations. Recent concerns surround the role pigs play in the emergence of diseases such Reston ebolavirus and H1N1 [1], [48]. Central Africa's forests are home to tens of thousands of wild pigs, including the Giant Forest Hog (*Hylochoerus meinertzhageni*) and the Red River Hog (*Potamochoerus porcus*), and are characterized as emerging disease hotspots [45]. Although no evidence has emerged supporting speculation of ebolavirus-associated wild pig die-offs in Africa, employing this assay in these species may address whether pigs are amplifiers, victims or carriers of the virus [48]. It is noteworthy that in the case of influenza pigs are considered “mixing vessels” for viruses and capable of generating new strains transmissible to humans [49]. The extensive bush meat trade in Africa provides ample opportunity for pathogen transmission from pigs to humans, and underlines the importance of disease surveillance in this species.

Wildlife managers frequently perform wide scale ecological surveys, simultaneously collecting biological samples and data on the density and distribution of wildlife. With the benefit of the new diagnostic capacity and sampling strategies described herein, different fecal sampling approaches can be integrated into these surveys to provide information that has thus far eluded us concerning the distribution, ecology and epidemiology of ebolavirus. For the first time, both the logistical and diagnostic capacities are available to immunologically screen large populations of wild great apes for previous exposure to ebolavirus and even estimate and monitor prevalence rates.

## Supporting Information

Figure S1 **Ebolavirus nucleoprotein sequences.** Sequence alignment of the nucleoprotein NP from *Zaire ebolavirus* (EBOV, Accession No. NP_066243), *Tai Forest ebolavirus* (TAFV, Accession No. ACI28629), *Reston ebolavirus* (RESTV, Accession No. BAB69003), *Sudan ebolavirus* (SUDV, Accession No. AAD51107) and *Bundibugyo ebolavirus* (BDBV, Accession No. ACI28620). The numbering of the amino acids is according to their position in the sequence. “*”, identical residues; “:” conserved residues; “.”, semi-conserved residues.
(PDF)
