# Supplementary material for: A New Approach for Monitoring Ebolavirus in Wild Great Apes
Source: PLoS Negl Trop Dis. 2014 Sep 18;8(9):e3143. doi: 10.1371/journal.pntd.0003143 (PMC4169258; doi:10.1371/journal.pntd.0003143)
Supplement: Figure S1 — Ebolavirus nucleoprotein sequences. Sequence alignment of the nucleoprotein NP from Zaire ebolavirus (EBOV, Accession No. NP_066243), Tai Forest ebolavirus (TAFV, Accession No. ACI28629), Reston ebolavirus (RESTV, Accession No. BAB69003), Sudan ebolavirus (SUDV, Accession No. AAD51107) and Bundibugyo ebolavirus (BDBV, Accession No. ACI28620). The numbering of the amino acids is according to their position in the sequence. “*”, identical residues; “:” conserved residues; “.”, semi-conserved residues. (PDF) [file pntd.0003143.s001.pdf]

Ebolaviruses nucleoprotein sequences

```
ZEBOV           MDSRPQKIWMAPSLTESDMDYHKILTAGLSVQQGIVRQRVIPVYQVNNLEEICQLIIQAF 60
CIEBOV          MESRAHKAWMTHTASGFETDYHKILTAGLSVQQGIVRQRVIQVHQVTNLEEICQLIIQAF 60
REBOV           MDRGTRRIWVSQNQGDTDLDYHKILTAGLTVQQGIVRQKIISVYLVDNLEAMCQLVIQAF 60
SEBOV           MDKRVRGSWALGGQSEVDLDYHKILTAGLSVQQGIVRQRVIPVYVVNDLEGICQHIIQAF 60
BEBOV           MDPRPIRTWMMHNTSEVEADYHKILTAGLSVQQGIVRQRIIPVYQISNLEEVCQLIIQAF 60
                *:      *        : **********:********::* *: : :** :** :****

ZEBOV           EAGVDFQESADSFLLMLCLHHAYQGDYKLFLESGAVKYLEGHGFRFEVKKRDGVKRLEEL 120
CIEBOV          EAGVDFQESADSFLLMLCLHHAYQGDYKQFLESNAVKYLEGHGFRFEVRKKEGVKRLEEL 120
REBOV           EAGIDFQENADSFLLMLCLHHAYQGDYKLFLESNAVQYLEGHGFKFELRKKDGVNRLEEL 120
SEBOV           EAGVDFQDNADSFLLLLCLHHAYQGDHRLFLKSDAVQYLEGHGFRFEVREKENVHRLDEL 120
BEBOV           EAGVDFQDSADSFLLMLCLHHAYQGDYKQFLESNAVKYLEGHGFRFEMKKKEGVKRLEEL 120
                ***:***:.******:**********:: **:*.**:*******:**:::::.*:**:**

ZEBOV           LPAVSSGKNIKRTLAAMPEEETTEANAGQFLSFASLFLPKLVVGEKACLEKVQRQIQVHA 180
CIEBOV          LPAASSGKSIRRTLAAMPEEETTEANAGQFLSFASLFLPKLVVGEKACLEKVQRQIQVHS 180
REBOV           LPAATSGKNIRRTLAALPEEETTEANAGQFLSFASLFLPKLVVGEKACLEKVQRQIQVHA 180
SEBOV           LPNVTGGKNLRRTLAAMPEEETTEANAGQFLSFASLFLPKLVVGEKACLEKVQRQIQVHA 180
BEBOV           LPAASSGKNIKRTLAAMPEEETTEANAGQFLSFASLFLPKLVVGEKACLEKVQRQIQVHA 180
                ** .:.**.::*****:******************************************:

ZEBOV           EQGLIQYPTAWQSVGHMMVIFRLMRTNFLIKFLLIHQGMHMVAGHDANDAVISNSVAQAR 240
CIEBOV          EQGLIQYPTAWQSVGHMMVIFRLMRTNFLIKFLLIHQGMHMVAGHDANDAVIANSVAQAR 240
REBOV           EQGLIQYPTAWQSVGHMMVIFRLMRTNFLIKYLLIHQGMHMVAGHDANDAVIANSVAQAR 240
SEBOV           EQGLIQYPTSWQSVGHMMVIFRLMRTNFLIKFLLIHQGMHMVAGHDANDTVISNSVAQAR 240
BEBOV           EQGLIQYPTSWQSVGHMMVIFRLMRTNFLIKFLLIHQGMHMVAGHDANDAVIANSVAQAR 240
                *********:*********************:*****************:**:*******

ZEBOV           FSGLLIVKTVLDHILQKTERGVRLHPLARTAKVKNEVNSFKAALSSLAKHGEYAPFARLL 300
CIEBOV          FSGLLIVKTVLDHILQKTEHGVRLHPLARTAKVKNEVNSFKAALSSLAQHGEYAPFARLL 300
REBOV           FSGLLIVKTVLDHILQKTDQGVRLHPLARTAKVRNEVNAFKAALSSLAKHGEYAPFARLL 300
SEBOV           FSGLLIVKTVLDHILQKTDLGVRLHPLARTAKVKNEVSSFKAALGSLAKHGEYAPFARLL 300
BEBOV           FSGLLIVKTVLDHILQKTEHGVRLHPLARTAKVKNEVSSFKAALASLAQHGEYAPFARLL 300
                ******************: *************:***.:*****.***:***********

ZEBOV           NLSGVNNLEHGLFPQLSAIALGVATAHGSTLAGVNVGEQYQQLREAATEAEKQLQQYAES 360
CIEBOV          NLSGVNNLEHGLFPQLSAIALGVATAHGSTLAGVNVGEQYQQLREAATEAEKQLQKYAES 360
REBOV           NLSGVNNLEHGLYPQLSAIALGVATAHGSTLAGVNVGEQYQQLREAATEAEKQLQQYAES 360
SEBOV           NLSGVNNLEHGLYPQLSAIALGVATAHGSTLAGVNVGEQYQQLREAATEAEKQLQQYAET 360
BEBOV           NLSGVNNLEHGLFPQLSAIALGVATAHGSTLAGVNVGEQYQQLREAATEAEKQLQKYAES 360
                ************:******************************************:***:

ZEBOV           RELDHLGLDDQEKKILMNFHQKKNEISFQQTNAMVTLRKERLAKLTEAITAASLPKTSGH 420
CIEBOV          RELDHLGLDDQEKKILKDFHQKKNEISFQQTTAMVTLRKERLAKLTEAITSTSLLKTGKQ 420
REBOV           RELDSLGLDDQERRILMNFHQKKNEISFQQTNAMVTLRKERLAKLTEAITLASRPNLGSR 420
SEBOV           RELDNLGLDEQEKKILMSFHQKKNEISFQQTNAMVTLRKERLAKLTEAITTASKIKVGDR 420
BEBOV           RELDHLGLDDQEKKILKDFHQKKNEISFQQTTAMVTLRKERLAKLTEAITSTSILKTGRR 420
                **** ****:**::** .*************.****************** :*  : . :

ZEBOV           YDDDDDIPFPGPINDDDNPGHQDDDPTDSQDTTIPDVVVDPDDGSYGEYQSYSENGMNAP 480
CIEBOV          YDDDNDIPFPGPINDNENSEQQDDDPTDSQDTTIPDIIVDPDDGRYNNYGDYPSETANAP 480
REBOV           QDDDNEIPFPGPISNNPDQDHLEDDPRDSRDTIIPNSAIDPEDGDFENYNGYHDDEVGTA 480
SEBOV           YPDDNDIPFPGPIYDDTHPNPSDDNPDDSRDTTIPGGVVDPYDDESNNYPDYEDSAEGTT 480
BEBOV           YDDDNDIPFPGPINDNENSGQNDDDPTDSQDTTIPDVIIDPNDGGYNNYSDYANDAASAP 480
                  **::******* :: .    :*:* **:** **.  :** *.   :* .* ..  .:.

ZEBOV           DDLVLFDLDEDDEDTKPVPNRSTKGGQQKNSQKGQHIEGRQTQSRPIQNVPGPHRTIHHA 540
CIEBOV          EDLVLFDLEDGDEDDHRPSSSSENNNKHSLTGTDSNKTSNWNRNPTNMPKKDSTQNNDNP 540
REBOV           GDLVLFDLDDHEDDNKAFELQDSSPQSQREIERERLIHPPPGNNKDDNRASDNNQQSADS 540
```

```
SEBOV           GDLDLFNLDDDDDDSRPGPPDRGQNKERAARTYGLQDPTLDGAKKVPELTPGSHQPGNLH 540
BEBOV           DDLVLFDLEDEDDADNPAQNTPEKNDRPATTKLRNGQDQDGNQGETASPRVAPNQYRDKP 540
                 ** **:*:: ::  .       .                              :

ZEBOV           SAPLTDNDRRNEPSGSTSPRMLTPINEEADPLDDADDETSSLPPLESDDEEQDRDGTSNR 600
CIEBOV          AQRAQEYARDNIQDTPTPHRALTPISEETGSNGHNEDDIDSIPPLESDEENNTETTITTT 600
REBOV           EEQEGQYNRHRGPERTTANRRLSPVHEEDTPIDQGDDDPSSPPPLESDDDDASSSQQDPD 600
SEBOV           ITKSGSNTNQPQGNMSSTLHSMTPIQEESEPDDQKDNDDESLTSLDSEGDEDGESISEEN 600
BEBOV           MPQVQDRSENHDQTLQTQSRVLTPISEEADPSDHNDGDNESIPPLESDDEGSTDTTAAET 600
                     .  .       :  : ::*: **  . .. :.: .* ..*:*: :

ZEBOV           TPTVAPPAPVYRDHSEKKELPQDEQQDQDHTQEARNQDSDNTQSEHSFEEMYRHILRSQG 660
CIEBOV          KNTTAPPAPVYRSNSEKEPLPQEKSQKQPNQVSGSENTDNKPHSEQSVEEMYRHILQTQG 660
REBOV           YTAVAPPAPVYRSAEAHEPPHKSSNEPAETSQLNEDPDIGQSKSMQKLGETYHHLLRTQG 660
SEBOV           TPTVAPPAPVYKDTGVDTNQQNGPSSTVDSQGSESEALPINSKKSSALEETYYHLLKTQG 660
BEBOV           KPATAPPAPVYRSISVDDSVPSENIPAQSNQTNNEDNVRNNAQSEQSIAEMYQHILKTQG 660
                  :.*******:.   .    .             :    :.:.   . * * *:*::**

ZEBOV           PFDAVLYYHMMKDEPVVFSTSDGKEYTYPDSLEEEYPPWLTEKEAMNEENRFVTLDGQQF 720
CIEBOV          PFDAILYYYMMTEEPIVFSTSDGKEYVYPDSLEGEHPPWLSEKEALNEDNRFITMDDQQF 720
REBOV           PFEAINYYHMMKDEPVIFSTDDGKEYTYPDSLEEAYPPWLTEKERLDNENRYIYINNQQF 720
SEBOV           PFEAINYYHLMSDEPIAFSTESGKEYIFPDSLEEAYPPWLSEKEALEKENRYLVIDGQQF 720
BEBOV           PFDAILYYHMMKEEPIIFSTSDGKEYTYPDSLEDEYPPWLSEKEAMNEDNRFITMDGQQF 720
                **:*: **::*.:**: ***..**** :*****  :****:*** ::::**:: ::.***

ZEBOV           YWPVMNHKNKFMAILQHHQ 739
CIEBOV          YWPVMNHRNKFMAILQHHK 739
REBOV           FWPVMSPRDKFLAILQHHQ 739
SEBOV           LWPVMSLRDKFLAVLQHD- 738
BEBOV           YWPVMNHRNKFMAILQHHR 739
                 ****. ::**:*:***.
```
